# The Effects of Preoperative Glycaemic Control (HbA1c) on Bariatric and Metabolic Surgery Outcomes: Data from a Tertiary-Referral Bariatric Centre in the UK

**DOI:** 10.1007/s11695-023-06964-x

**Published:** 2024-01-15

**Authors:** Rebekah Wilmington, Mahmoud Abuawwad, Guy Holt, Robyn Anderson, Rami Aldafas, Sherif Awad, Iskandar Idris

**Affiliations:** 1 Clinical, Metabolic and Molecular Physiology Research Group, MRC-Versus Arthritis Centre for Musculoskeletal Ageing Research, University of Nottingham, Royal Derby Hospital Centre, Uttoxeter Road, Derby, DE22 3NE UK; 2 National Institute for Health Research (NIHR) Nottingham Biomedical Research Centre, Nottingham, UK; 3 East Midlands Bariatric & Metabolic Institute (EMBMI), Royal Derby Hospital, University Hospitals of Derby & Burton NHS Foundation Trust, Derby, UK; 4 Faculty of Public Health, College of Health Science, The Saudi Electronic University, Riyadh, Saudi Arabia

**Keywords:** Bariatric surgery, Type 2 diabetes, Glycated haemoglobin, HbA1c, Peri-operative, Post-operative, Complications, Mortality, Intensive care, Length of stay

## Abstract

**Background:**

Current recommendations advocate the achievement of an optimal glucose control (HbA1c < 69 mmol/mol) prior to elective surgery to reduce risks of peri- and post-operative complications, but the relevance for this glycaemic threshold prior to Bariatric Metabolic Surgery (BMS) following a specialist weight management programme remains unclear.

**Methods:**

We undertook a retrospective cohort study of patients with type 2 diabetes mellitus (T2DM) who underwent BMS over a 6-year period (2016–2022) at a regional tertiary referral following completion of a specialist multidisciplinary weight management. Post-operative outcomes of interest included 30-day mortality, readmission rates, need for Intensive Care Unit (ICU) care and hospital length of stay (LOS) and were assessed according to HbA1c cut-off values of < 69 (*N* = 202) and > 69 mmol/mol (*N* = 67) as well as a continuous variable.

**Results:**

A total of 269 patients with T2D were included in this study. Patients underwent primary Roux en-Y gastric bypass (RYGB, *n* = 136), Sleeve Gastrectomy (SG, *n* = 124), insertion of gastric band (*n* = 4) or one-anastomosis gastric bypass (OAGB, *n* = 4). No significant differences in the rates of complications were observed between the two groups of pre-operative HbA1c cut-off values. No HbA1c threshold was observed for glycaemic control that would affect the peri- and post-operative complications following BMS.

**Conclusions:**

We observed no associations between pre-operative HbA1C values and the risk of peri- and post-operative complications. In the context of a specialist multidisciplinary weight management programme, optimising pre-operative HbA1C to a recommended target value prior to BMS may not translate into reduced risks of peri- and post-operative complications.

**Graphical Abstract:**

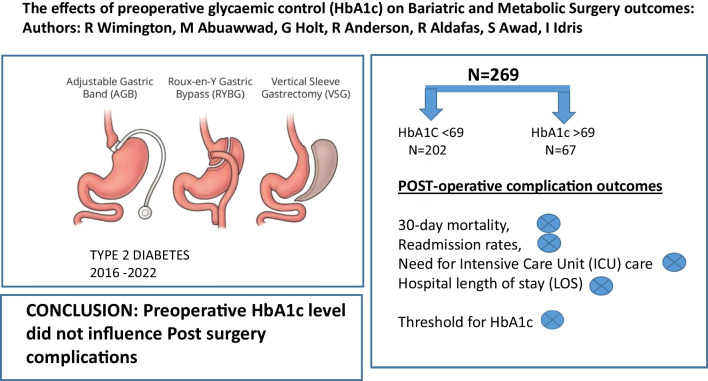

## Introduction

Bariatric and metabolic surgery (BMS) is increasingly recognised to be the most effective intervention to induce and maintain significant weight loss amongst patients living with class 2 obesity or above (BMI > 35 kg/m [2]). Amongst patients who have obesity-related comorbidities [1, 2], pre-operative optimisation of medical comorbidities is considered crucial in reducing short- and long-term complications of BMS.

Type 2 diabetes (T2D) is present in approximately one-third of patients undergoing BMS [3]. Elevated pre-operative HbA1c level (a measure of chronic hyperglycaemia), typically at > 8% (58 mmol/mol), has been reported to increase the risks of postoperative complications, but this evidence was largely derived from studies involving patients undergoing non-bariatric surgery procedures [4–10]. Nevertheless, several consensus statements have advocated postponement of elective surgery, which include BMS, until optimal HbA1c levels were achieved [11, 12]. The National Institute for Health and Care Excellence (NICE) along with the Association of Anaesthetists of Great Britain and Ireland advocate referral to diabetes specialist teams when HbA1c is ≥ 8.5% (69 mmol/L) [13], whilst the Society for Ambulatory Anaesthesia (SAMBA) recommends a threshold HbA1c level of 7.0% (53 mmol/L) [14].

Achievement of these ‘optimal’ HbA1c values can, however, be challenging for patients undergoing BMS, not least because bariatric surgical options are often sought for patients with poorly controlled HbA1c levels with the aim of improving glycaemic control and inducing diabetes remission [15, 16]. Furthermore, the premise of BMS is not only to induce weight loss but rapid amelioration of obesity-related comorbidities and metabolic complications driven by high HbA1c levels [17, 18]. Several studies undertaken on BMS patients report that elevated HbA1c does not lead to increased postoperative morbidity or mortality in obese patients with diabetes [19–25]. However, many of these studies assess gastric bypass surgery [21, 22, 24] and had limitations such as using HbA1c as a categorical variable, not examining an optimal HbA1c cut-off, not adjusting for important baseline characteristics, not including ICU admissions as an outcome and not including input from a specialist multi-disciplinary team prior to BMS. We therefore sought to report the effects of preoperative HbA1c level on peri-operative outcomes of BMS within the setting of a tertiary referral Bariatric Centre with a view to defining if there is an optimal HbA1c cut-off level and to whether elective BMS should be delayed in patients with elevated HbA1c levels.

## Methods

This was a retrospective cohort study of obese patients with type 2 diabetes mellitus who underwent BMS over a 6-year period (2016–2022) at the East Midlands Bariatric Metabolic Institute, Royal Derby Hospital, a regional tertiary referral centre that serves a population of 3.2 million adults. All patients completed a 6- to 12-month tier 3 specialist multidisciplinary weight management programme prior to surgery. Exclusion criteria included the following: patients with type 1 diabetes mellitus (as this is not a recognised obesity-related comorbidity) and patients undergoing revisional BMS. All patients underwent a minimum of 2 weeks and a maximum of 4 weeks pre-operative diet supervised by our dietitians.

Collected data included demographic details, anthropometric measurements, laboratory assessments, operation notes, referral and follow-up letters. Post-operative outcomes of interest included 30-day mortality, readmission rates, need for Intensive Care Unit (ICU) care and hospital length of stay (LOS). Surgical complication rates at grades 4 and 5 of the Clavien-Dindo system was collected (although our data was not able to provide adequate information for grades 1 to 3 complications for many patients due to incomplete data recording in nursing and medical notes going back to 2016). There were no missing data for all outcomes we were investigating.

### Statistical Analysis

Continuous data were analysed using the independent sample *t* test, whilst categorical data were analysed using Chi-square. Data were presented as mean ± standard deviation (SD). Non-parametric data were analysed using Mann–Whitney and presented as median ± interquartile range (IQR). For dichotomous variables, the chi-squared test was used when values in all cells were greater than five. Otherwise, the *p*-value for the Fisher’s exact test was used. Simple logistic regression was applied between the HbA1c level with combined risk of complications. Patients were divided into two groups: Group 1 (HbA1c of < 69 mmol/mol) and Group 2 (HbA1c ≥ 69 mmol/mol) to reflect current guidance surrounding the HbA1c thresholds and allow assessment between pre-operative glycaemic control and complications. Statistical analyses were performed using STATA version 17 software, and significance was accepted at *p* = 0.05 level.

## Results

A total of 269 patients with T2D were included in this study. All patients received tier-3 medical weight management intervention until they were ready to proceed with BMS. Patients underwent primary Roux en-Y gastric bypass (RYGB, *n* = 136), Sleeve Gastrectomy (SG, *n* = 124), insertion of gastric band (*n* = 4) or one-anastomosis gastric bypass (OAGB, *n* = 4). All procedures were performed laparoscopically. Baseline demographics comparing the two groups are given in Table 1.

**Table 1:** Demographic variables in patients with an HbA1c < 69 mmol/mol and HbA1c ≥ 69 mmol/mol

	Group 1	Group 2	p-value
	HbA1c < 69 (n = 202)	HbA1c ≥ 69 (n = 67)	
Age (years)	56 ± 10.8	55.2 ± 10.3	0.54
Sex, female	142(76.3%)	44(23.7%)	0.48
Preoperative body mass index (kg/m^*2*^)	50.7 ± 29.6	48.6 ± 10.4	0.61
Preoperative Haemoglobin A1C mmol/mol	49.3 ± 9.4	84.7 ± 14.9	< 0.000^*^
*Procedure type*			0.91
*Laparoscopic sleeve gastrectomy*	93(75%)	31(25%)	
*Laparoscopic insertion of gastric band*	4(100%)	0(0%)	
*Laparoscopic Roux en-Y gastric bypass*	101(74.3%)	35(25.7%)	
*One-anastomosis gastric bypass*	4(80%)	1(20%)	
Heart failure	10(62.5%)	6(37.5%)	0.23
Coronary artery disease	14(60.9%)	9(39.1%)	0.09
Cerebrovascular disease	5(62.5%)	3(37.5%)	0.41
Nephropathy	9(81.8%)	2(18.9%)	0.73
Neuropathy	10(58.8%)	7(41.2%)	0.11
Obstructive sleep apnoea	87(77%)	26(23%)	0.54
Hypertension	127(75.1%)	67(24.9%)	0.80
Chronic obstructive pulmonary disease	18(78.3%)	5(21.7%)	0.71
Insulin use	39(58.2%)	28(41.8%)	< 0.000^*^
ACE inhibitors and receptor blockers	103(79.2%)	27(20.8%)	0.12
Other antihypertensive	70(80.5%)	17(19.5%)	0.16
Aspirin	20(76.9%)	6(23.1)	0.82
Warfarin			
	4(50%)	4(50%)	0.10

^∗^Statistical significance (*p* ≤ 0.05)

In group 1 (*n* = 202), the majority of BMS patients were women (mean ± SD age, preoperative BMI and HbA1c were 56 ± 10.8 years, 50.7 ± 29.6 kg/m^2^ and 49.3 ± 9.4 mmol/mol, respectively). In group 2 (*n* = 67), the majority of BMS patients were male (mean ± SD age, preoperative BMI and HbA1c were 55.2 ± 10.3 years, 48.6 ± 10.4 kg/m^2^ and 84.7 ± 14.9 mmol/mol, respectively. No significant differences were noted between groups (Table 2).

**Table 2:** Postoperative outcomes in Group 1 and Group 2

Outcomes	HbA1c < 69 mmol/mol (*N* = 202)	HbA1c > 69 mmol/mol (*N* = 67)	*p-value*
Mortality	2	0	1.00
(Grade 5 Clavien-Dindo)			
Grade 4 Clavien-Dindo	0	0	NS
30-day readmission	8	3	1.00
ICU admission	5	3	0.416
Length of Stay	2	2	0.7936

Peri-operative outcomes including complications, mortality, 30-day readmission, ICU admission and LOS are listed in Table 2. There were no significant differences between the groups. Similarly, no differences were found for the combined risk of complications between groups of HbA1c levels (Table 3). No HbA1c threshold was observed for glycaemic control that would affect the outcomes of BMS.

**Table 3:** Association between HbA1c levels and combined adverse outcomes

Combined outcome			
*Predictors*	*Odds Ratios*	*CI*	*p*
Intercept	0.06	0.01–0.29	< 0.001
HbA1c mmol/mol	1.00	0.97–1.03	0.858
Observations	269		
*R*^2^ Tjur	0.000		

The unadjusted odds ratio for HbA1c levels was 1.00 (95% CI, 0.97–1.03), indicating no significant association with the occurrence of the adverse combined outcome

## Discussion

Optimising pre-operative HbA1c levels is an important component of preparation for BMS. However, in selected patients, reductions of HbA1c to < 69 mmol/mol may be challenging. This study in patients living with type 2 diabetes found no statistical difference in LOS stay, ICU admissions, 30-day hospital readmissions or mortality after BMS when comparing preoperative HbA1c values ≥ 69 vs < 69 mmol/mol. Similarly, no threshold for HbA1c level was identified that would influence adverse BS outcomes in patients undergoing BMS. Our data included 27 patients whose HbA1c is > 86 mmol/mol (10%), 12 of whom have HbA1c levels of > 100 mmol/mol (11.3%). Our findings suggest that in the context of a multi-disciplinary physician-supported bariatric surgery service (which also included psychology and dietetic input), BMS can be performed safely in patients with elevated HbA1c levels. Whilst this study is supportive of others undertaken in BMS patients [19–25], our study addresses previous limitations by including patients who underwent a minimum of 6-months pre-operative MDT input, adjusting for additional confounders and including ICU admission as an outcome measure.

Previous studies in non-BMS patients [4–10] have provided guidelines and recommendations to achieve ‘optimal’ HbA1c cut-off of < 69 mmol/mol prior to elective surgery, but these findings have not been supported in studies undertaken on BMS patients. Several reasons may explain the discordance between studies in BMS and non-BMS patients. Unlike non-BMS operations, BMS is known to induce significant reductions in glucose levels prior to and after surgery [1, 2]. Improvements in glucose levels are largely driven by the pre-operative liver shrinkage diet which are routinely required for all patients prior to BMS, occasionally in the form of a very low-calorie diet programme as well as other post-BMS metabolic benefits which have been reported to occur independent of weight loss [26]. Furthermore, previous experimental data, albeit in animal studies, have shown that calorie restriction can modulate the physiologic stress response to surgical injury, which likely underlies peri-operative complications, enhance recovery of renal function [27] and mitigate hepatic damage [28] after surgical ischemia–reperfusion injury.

Limitations of this study include retrospective data collection and residual confounding by not including data on Apnoea Hypopnea Index, ethnicity, medications, 24-h blood pressure measurements and relatively small number of patients. In addition, specific surgical complications such as wound infection, need for blood transfusion and the use of anti-emetics are not fully available for all patients going back to 2106 such that data collection for grades 1 to 3 complications via the Clavien-Dindo system is not possible. Similarly, data for pre-operative and post-operative glucose, the use of sliding scale insulin and anti-diabetic therapy included multiple missing data for many patients since 2016 and therefore not included in our analysis. In addition, HbA1c level was monitored locally by individual primary care practices at 6 to 12 months post-operation and therefore not available within our database.

In conclusion, our data suggest that pre-operative HbA1c has limited utility in the decision whether to proceed with BMS and no effect on operative outcomes. Provided patients who are suitable for bariatric surgery undergo a patient-specific integrated multi-disciplinary approach prior to BMS with the aim of co-morbidity optimisation; the arbitrary HbA1c threshold of < 69 mmol/mol should not be used as a basis to exclude or delay patients from having bariatric surgery. Strategies to further optimise HbA1c level prior to surgery include the use of extended liver shrinkage diets pre-operatively, as is commonplace in our Centre. Additionally, detailed preoperative counselling of patients with regard to the risks and benefits of proceeding with BMS, in the context of suboptimal glycaemic control, versus delaying BMS whilst awaiting further glycaemic optimisation is encouraged.
